# Digital Biomarkers for the Objective Assessment of Disability in Neurogenic Thoracic Outlet Syndrome

**DOI:** 10.3390/s21227462

**Published:** 2021-11-10

**Authors:** Bijan Najafi, Mohsen Zahiri, Changhong Wang, Anmol Momin, Paul Paily, Bryan M. Burt

**Affiliations:** 1 Division of Vascular and Endovascular Surgery, Michael E. DeBakey Department of Surgery, Baylor College of Medicine, Houston, TX 77030, USA; Bijan.Najafi@bcm.edu (B.N.); mohsen_z1579@yahoo.com (M.Z.); changhong.wang@bcm.edu (C.W.); anmol.momin@bcm.edu (A.M.); 2 Department of Physical Medicine and Rehabilitation, Baylor College of Medicine, Houston, TX 77030, USA; paul.paily@bcm.edu; 3 Division of Thoracic Surgery, Michael E. DeBakey Department of Surgery, Baylor College of Medicine, Houston, TX 77030, USA

**Keywords:** thoracic outlet syndrome, wearables, sensors, disability, digital health, upper extremity

## Abstract

Neurogenic thoracic outlet syndrome (nTOS) is a musculoskeletal disorder in which compression of the brachial plexus between the scalene muscles of the neck and the first rib results in disabling upper extremity pain and paresthesia. Currently there are no objective metrics for assessing the disability of nTOS or for monitoring response to its therapy. We aimed to develop digital biomarkers of upper extremity motor capacity that could objectively measure the disability of nTOS using an upper arm inertial sensor and a 20-s upper extremity task that provokes nTOS symptoms. We found that digital biomarkers of slowness, power, and rigidity statistically differentiated the affected extremities of patients with nTOS from their contralateral extremities (n = 16) and from the extremities of healthy controls (n = 13); speed and power had the highest effect sizes. Digital biomarkers representing slowness, power, and rigidity correlated with patient-reported outcomes collected with the Disabilities of the Arm, Shoulder and Hand (DASH) questionnaire and the visual analog scale of pain (VAS); speed had the highest correlation. Digital biomarkers of exhaustion correlated with failure of physical therapy in treating nTOS; and digital biomarkers of slowness, power, and exhaustion correlated with favorable response to nTOS surgery. In conclusion, sensor-derived digital biomarkers can objectively assess the impairment of motor capacity resultant from nTOS, and correlate with patient-reported symptoms and response to therapy.

## 1. Introduction

Neurogenic thoracic outlet syndrome (nTOS) is a musculoskeletal disorder in which the brachial plexus is dynamically compressed within the scalene triangle, an anatomic space bordered by the anterior and middle scalene muscles where they insert on the first rib. The etiology of nTOS is not well understood; however, it is believed to be a consequence of traumatic or repetitive scalene muscle injury that narrows the scalene triangle. Brachial plexus compression results in upper extremity pain and paresthesia that are exacerbated by the organic narrowing of the scalene triangle that occurs with hand-over-head activity. Neurogenic TOS is estimated to affect 3–80/1000 individuals, is highly prevalent among industrial workers, athletes, computer users, and musicians [1,2,3,4,5], and accounts for substantial loss of employment and inability to perform activities of daily living (ADL) [6,7,8,9].

Patients with nTOS are generally first treated with physical therapy (PT) to relieve muscular compression of the brachial plexus. Surgical decompression with first rib resection and scalenectomy is offered if PT is ineffective. However, there are no standardized methods for assessing the severity of nTOS or for assessing the efficacy of any therapy for nTOS. This has resulted in highly variable nTOS treatment protocols and has perpetuated confusion among patients and providers on assignment of best therapy. For example, PT is ineffective in the majority of cases and commonly aggravates the symptoms of nTOS, yet individuals with nTOS often undergo long unproductive periods of PT before being offered surgery [10,11,12,13,14,15,16]. Further, PT is prescribed as a variety of regimens, and at least seven approaches to first rib resection and scalenectomy are currently practiced. The lack of reliable metrics for assessing the severity of nTOS has precluded the rigorous assessment of nTOS therapy, limited confidence in available therapies, and constrained the design and conduct of the clinical trials that are needed to advance the field.

We and others have investigated patient-reported outcome measures (PROMs) for the semi-quantitative assessment of nTOS severity, including the Disabilities of the Arm, Shoulder and Hand (DASH) questionnaire and other questionnaires for pain and quality of life [6,17,18,19,20,21]. These instruments are subjective, however, and suffer from the selection and scale perception biases inherent in self-reporting modalities [22]. Further, there is no PROM designed specifically for nTOS, and PROMs do not capture the extremity-specific physiologic limitations of nTOS. To address these challenges, we extracted sensor-based metrics of extremity function during a brief hand-over-head extremity task that narrows the scalene triangle and provokes the symptoms of nTOS. Our primary goal was to identify digital biomarkers of upper extremity function that are impacted by nTOS and that can be applied to objectively define its severity and monitor its response to therapy.

## 2. Materials and Methods

**Participants.** Eligible participants with nTOS were prospectively recruited from the Baylor College of Medicine TOS Clinic in accordance with an approved IRB protocol (H-38994). Inclusion criteria included age ≥18 years and diagnosis of unilateral nTOS. Exclusion criteria were bilateral nTOS, prior surgical treatment for nTOS, and concomitant pectoralis minor syndrome. To be assigned the diagnosis of nTOS, patients were required to satisfy four of four Society of Vascular Surgeons (SVS) diagnostic criteria [14]:

(1) Central findings including symptoms of irritation/inflammation at the scalene triangle and/or scalene muscle tenderness.
(2) Peripheral findings including upper extremity symptoms of central nerve compression (numbness, pain, weakness, etc.) at baseline or with provocative maneuvers.
(3) Absence of other diagnoses responsible for the majority of symptoms, in accordance with a standard clinical evaluation that we have described previously [23].
(4) Response to ultrasound-guided scalene muscle block [24], considered as ≥30% subjective improvement in the predominant nTOS symptom.

Healthy subjects ≥18 years old without a history of pain, surgery, or limitation of the upper extremity were prospectively recruited to our IRB-approved protocol and included students, research interns, and staff at our institution.

**nTOS Therapy**. All nTOS patients were initially treated conservatively with a standardized outpatient PT regimen based on the Edgelow protocol [25] for at least 8 weeks. A sub-sample of participants included those whose symptoms did not meaningfully improve with PT and who desired and underwent surgery. Meaningful symptom improvement by PT was a clinical decision made between the physician team, patient, and physical therapist. This represents the current clinical standard for determining response to PT and underscores the rationale for this study. Surgery for nTOS was performed by transthoracic robotic first rib resection, scalenectomy, and brachial plexus neurolysis, an approach that we have pioneered and that results in minimal surgical morbidity [26,27].

**Figure 1:**
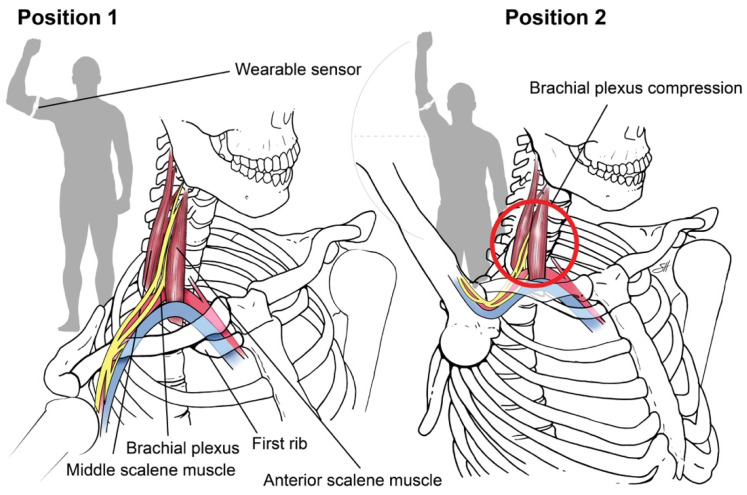
**Press Test**. We designed a 20-s repetitive hand-over-head exercise that narrows the scalene triangle and exacerbates the symptoms of nTOS by anatomically narrowing the scalene triangle with arm elevation. Using an inertial measurement unit, digital biomarkers of nTOS were identified. Copyright 2020 Baylor College of Medicine.

**Sensor-based assessment of extremity function.** We designed a “Press Test” to assess the limitations of upper extremity motor capacity from nTOS using wearable sensors. The Press Test was modeled after the elevated arm stress test (EAST), a provocative clinical test that narrows the scalene triangle and exacerbates the prototypic symptoms of nTOS as a result of hand-over-head activity [14]. An inertial measurement unit (IMU) (LEGSys™, BioSensics, Newton, MA, USA) was placed on the upper arm using an adjustable strap. Each IMU includes a tri-axial accelerometer and a tri-axial gyroscope that enable continuous, wireless measurements of upper-arm acceleration and rotation speed at high temporal resolution (100 Hz). Sensors of the IMU were oriented to a set reference frame by axis correction using quaternion algorithms [28,29]. A single research assistant performed the test on each subject, who begins with the upper extremity abducted 90 degrees with the elbow flexed 90 degrees (position 1, Figure 1).

The subject then completed 180-degree arm abduction with elbow extension to the “stick-up” position (position 2, Figure 1) and then returned to position 1, repeating the cycle as rapidly as possible for 20 s. At the conclusion of the test, we extracted 3D angles, angular velocity, and position parameters according to our prior methods [28,30]. A simple moving average filter (6 points) was applied to reduce artifacts, and zero crossing (ZC) points which did not satisfy minimum expected time-interval thresholds excluded [31]. Matlab software (Mathwork, Inc., Ver R2018a) was used for signal pressing and feature extraction.

**Patient-reported outcome measures (PROMs).** Demographic information including age, gender, height, weight, body mass index (BMI), hand dominance, and ethnicity was collected at enrollment. Before undergoing the Press Test, participants completed the DASH questionnaire, an instrument used to measure functional disability and QOL in individuals with musculoskeletal conditions of the upper extremity, and which we have used to measure patient-reported disability in nTOS [17]. The DASH uses 11 items to measure physical function and symptoms relevant to upper extremity disability and calculates a score from 0 (asymptomatic) to 100 (totally incapacitated). We captured patient-reported pain using the visual analog scale (VAS) of pain, which ranges 0–10 [32]. Other assessments included depression (Center for Epidemiologic Studies Depression (CES-D) scale [33]), activities of daily living (Barthel Index), work disability (patient reporting), use of narcotic and/or non-narcotic pain medication, and presence of headache associated with upper extremity symptoms. A DASH score ≥85 was considered severe. A CES-D score of ≥16 identified depression [33]. A Barthel Index ≤90 was considered moderate/total dependence.

**Statistical analysis.** Continuous variables were expressed as mean ± standard deviation (SD). Unpaired t-tests, Mann–Whitney U-tests, or Chi-squared tests were used according to the scale of the investigated variable and the distribution of data. Repeated measures ANOVA tests compared affected and unaffected extremities within the nTOS group, and dominant and non-dominant extremities in controls. ANCOVA tests were used for comparisons between nTOS and control groups with adjustment for age and BMI. Cohen’s effect sizes (‘d’) were determined to compare variables of interest, where d of 0.2–0.49 was small, 0.5–0.79 was moderate, 0.8–1.29 was large, and ≥1.3 was very large. Spearman correlation coefficients (‘Rho’) were used to examine association of sensor features and DASH. To determine the optimum cut point to distinguish between nTOS cases and healthy control subjects, we applied receiver operating characteristic (ROC) curve analysis for the digital biomarker with the largest effect size. Sensitivity, specificity, and area under curve were reported for the digital biomarker with the largest effect size for distinguishing between nTOS cases and healthy controls. Statistical significance was defined at *p <* 0.050, and all statistical analyses were performed using SPSS software (v26; IBM).

## 3. Results

### 3.1. Patient Sample

Sixteen participants with nTOS and 13 age-matched control subjects were enrolled. Table 1 displays the demographic and clinical characteristics of participants. No statistical significance between nTOS and control participants was observed for age, height, and hand dominance; however, the nTOS group participants were enriched for females and for individuals with higher BMI.

Average duration of nTOS diagnosis was 3.6 ± 4.9 years. In 63% of cases, nTOS affected the dominant extremity. DASH scores and Barthel indices were significantly worse in the nTOS group, among which 44% had depression, 25% used narcotics, 38% had moderate/total dependence, 75% had moderate/severe limitations in ADL, 31% had severe extremity disability (DASH ≥ 85), and 25% reported work disability.

### 3.2. Sensor-Derived Assessment of Extremity Function

**Figure 2:**
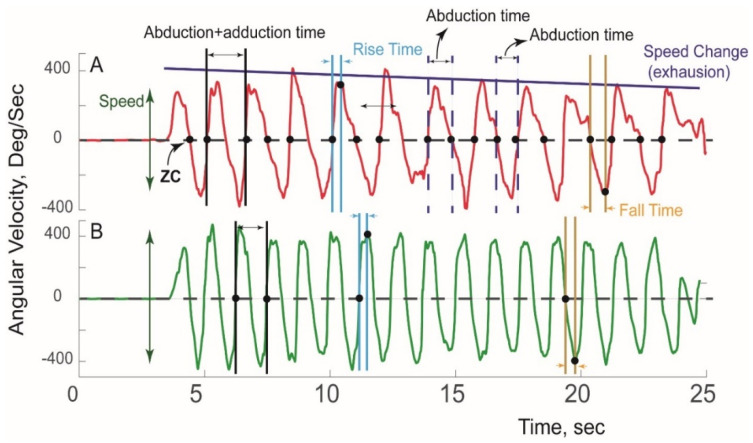
**Sensor-derived assessment of extremity function.** A typical pattern of angular velocity measured during a Press Test is shown for the (**A**) affected extremity and (**B**) unaffected extremity in a patient with unilateral nTOS.

A typical pattern of sensor-derived angular velocity of the affected and unaffected extremity of an nTOS patient during a Press Test is illustrated in Figure 2.

Using ZC and peak detection algorithms, 18 kinetic and kinematic features of five categories of motor capacity (slowness, weakness, rigidity, exhaustion, and unsteadiness) were extracted (Table 2) using our previously validated algorithms [34,35,36]. Digital biomarkers of slowness included speed (average range of angular velocity), duration of abduction+adduction, rise time (duration of abduction acceleration), fall time (duration of adduction acceleration), abduction time (duration from Position 1→2, Figure 1), adduction time (duration from Position 2→1), and total number of cycles completed. To estimate weakness, power was calculated as the product of range of angular velocity and range of angular acceleration. To determine rigidity, range of abduction/adduction rotation was calculated using quaternion and Kalman filters as we have described [28]. Each variable was determined for each cycle, and their averages were compared between groups. Exhaustion was determined by comparing change in features (including speed, rise time, power) in the first and last 10 s. Unsteadiness was quantified using coefficients of variation for metrics indicative of slowness, power, and rigidity.

### 3.3. Digital Biomarkers of nTOS Severity

All participants were able to complete the Press Test. Six digital biomarkers were statistically different between the affected and unaffected extremity of nTOS patients (Table 3).

**Figure 3:**
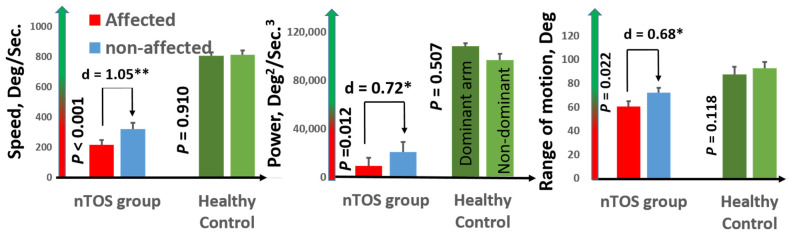
**Digital biomarkers of nTOS severity.** Digital biomarkers of speed, power, and range of motion statistically differentiated the affected and unaffected extremities of 16 nTOS patients. Each of these biomarkers in the affected (and the unaffected) extremity in nTOS patients was statistically decreased compared with a healthy control population (*p* < 0.05, * moderate effect size; ** large effect size).

Speed (a measure of slowness) and power (a measure of weakness) had the largest effect sizes for distinguishing the affected and unaffected extremities of nTOS patients (d = 1.05 and d = 0.91; *p* < 0.001 and *p* = 0.039, respectively). Figure 3 graphically illustrates comparisons of speed and power, as well as range of motion (a digital biomarker of rigidity) between the affected and unaffected extremities of nTOS patients. No digital biomarkers of exhaustion or steadiness were statistically different between nTOS-affected and unaffected extremities.

In the control group, no difference was observed between the dominant and non-dominant extremities for any digital biomarkers (*p* > 0.050, d = 0.02–0.25). Each of the nine digital biomarkers of slowness, weakness, and rigidity was statistically different between nTOS-affected extremities and dominant control extremities, with very large effect sizes (d = 1.37–5.0, *p* < 0.050). Interestingly, these same nine digital biomarkers were also statistically different between the contralateral extremity of nTOS patients and the dominant (and also non-dominant) extremities of controls (*p* < 0.050, d = 0.89–4.1), suggesting that the disability of unilateral nTOS can extend to the “unaffected” extremity. None of the nine digital biomarkers of exhaustion and steadiness statistically differentiated the affected (or unaffected) extremities of nTOS patients from healthy control extremities. Because the control group was enriched for males compared with the nTOS group, we performed a sensitivity analysis comparing digital biomarkers between the five males and eight females in the control group and found no statistical differences in any of the 18 digital biomarkers in this study. Using ROC curve analysis, we found that a cutoff for speed of 488.6 deg/s had 100% sensitivity and specificity to distinguish between nTOS from healthy control subjects (AUC = 1). To distinguish between non-affected nTOS and healthy controls, a cutoff of 514.2 deg/sec had 100% sensitivity and 95% specificity (AUC = 0.996).

### 3.4. Digital Biomarkers and PROMs

**Figure 4:**
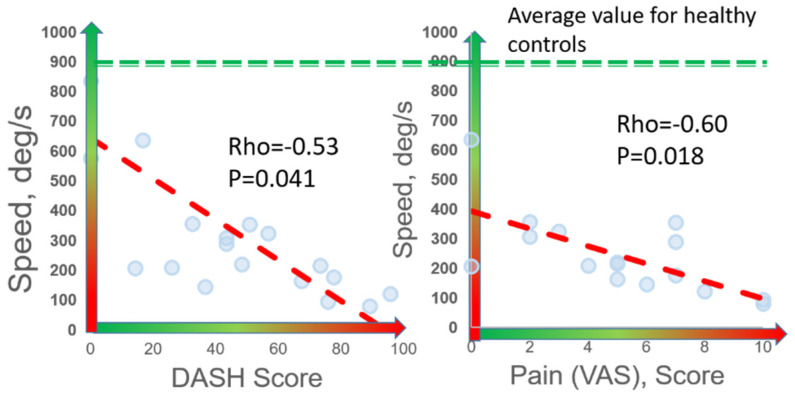
**Correlation of digital biomarkers with PROMs.** Among 16 nTOS patients, 7 digital biomarkers (shown here is speed) correlated with patient-reported disability (DASH) and pain (VAS).

Speed was the digital biomarker with the highest correlation to both DASH (rho = −0.53, *p* = 0.041) and VAS (rho = −0.60, *p* = 0.018) (Figure 4). Significant correlations were observed between DASH and other digital biomarkers of slowness (abduction time, rise time, abduction+adduction time, and #cycles), rigidity, and weakness (rho = 0.42–0.53, *p* < 0.050). Significant correlations were observed between VAS and these same digital biomarkers of slowness, weakness, and rigidity (rho = 0.49–0.62, *p* < 0.050). Interestingly, patients reporting work disability (n = 4) and those that did not report work disability (n = 12) were statistically differentiated by digital biomarkers of speed (*p* = 0.044), rigidity (*p* = 0.034), weakness (*p* = 0.021), and exhaustion (*p* = 0.045), whereas no other clinical, demographic, or PROM variables were statistically different between these two groups (*p* > 0.050).

### 3.5. Digital Biomarkers for Predicting Response to PT

Of the 16 patients in our sample, eight (50%) experienced meaningful symptom improvement and eight (50%) did not. Aside from pre-treatment DASH scores, which were worse in non-responders (71.0 ± 20.0) compared with responders (45.5 ± 23.1, *p* = 0.023), no clinical or demographic variables statistically differentiated these groups. Although not statistically significant, those who did not respond to PT were older (40.1 ± 12.6 years for non-responders vs. 32.9 ± 11.1 years for responders, *p* = 0.2), had higher pain scores (VAS 5.9 ± 3.1 in non-responders vs. 4.4 ± 2.8 in responders, *p* = 0.3), and had worse symptoms of depression by the CES-D scale (22.8 ± 16.4 in non-responders vs. 15.5 ± 5, *p* = 0.4). Digital biomarkers indicating exhaustion of the affected arm were significantly different between responders and non-responders and included decline in power (20.8% in non-responders vs. 2.7% in responders, *p* = 0.048) and increase in rise time (27% in non-responders vs. 5% in responders, *p* = 0.02).

### 3.6. Digital Biomarkers for Monitoring Response to nTOS Surgery

**Figure 5:**
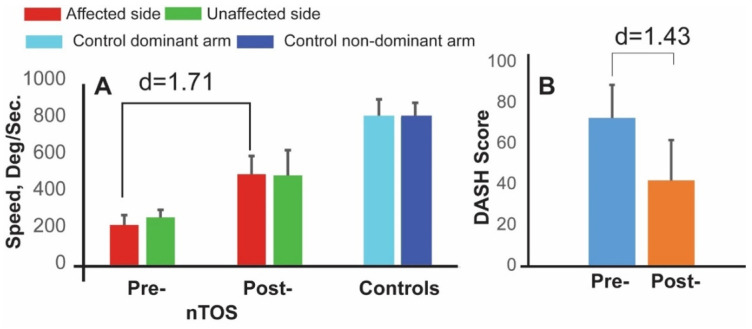
**Digital biomarkers for monitoring response to nTOS therapy.** Digital biomarker of speed (**A**) and DASH scores (**B**) are shown at preoperative and 4 week-postoperative time points for 4 nTOS patients undergoing surgery.

The subset of four patients who underwent surgery participated in follow-up assessments that included a repeat Press Test 1 month later. At this time, each of the 18 digital biomarkers statistically improved compared with their preoperative values. Biomarkers with the greatest magnitude of change (large to very large effect sizes) were improvements in speed and in power, as well a decrease in a biomarker of exhaustion (the rate of decline in power). Speed was the dominant parameter, increasing 121% on average following surgery with a very large effect size (d = 1.71, *p* < 0.050, Figure 5). DASH scores also improved after surgery but with lesser magnitude and effect size (42%, d = 1.43, *p* < 0.050). Interestingly, digital biomarkers of speed, power, and exhaustion on the contralateral side also improved at 4 weeks following surgery. The largest effect size (d = 1.67, *p* < 0.050) was observed for speed, and this is illustrated in Figure 5.

## 4. Discussion

Neurogenic TOS is a debilitating condition. Seventy-five percent of the patients in our cohort had at least moderate limitation in ADL, approximately half were depressed, and one quarter reported work disability. The management of patients with nTOS is controversial, and standardized treatment guidelines for nTOS have not been established. This is due largely to the absence of objective metrics of nTOS severity to inform clinical decision-making, monitor treatment outcomes, and evaluate new therapies. A monitoring platform that objectively assesses function of the affected extremity would provide especially meaningful data for the initial and ongoing assessment of patients with nTOS.

The prevailing mode of assessing disability in nTOS is the subjective impression of the treating physician, and prior reports in the field relied on outcomes using provider estimates of “excellent/good/fair/poor” results [9,13,15,24,37,38,39,40,41,42,43,44,45,46,47,48,49,50], often referenced as “Derkash’s classification” [51]. Such models of practitioner-dependent reporting suffer from observer and confirmation biases that are highlighted by studies in which patients with favorable outcomes following nTOS therapy were subsequently judged to not have benefited when reviewed by an independent observer [52,53]. More recently, a limited number of different groups have investigated PROMs to assess the response to surgical intervention for nTOS [6,17,18,19,20,21]; however, PROMs are also limited by their subjective nature and by the scale and perception biases inherent in the survey platform. Further, there are no PROMs designed specifically for nTOS, and available patient-reported questionnaires may not appropriately capture the disability of this condition.

We designed a simple 20-s Press Test that narrows the scalene triangle and limits upper extremity function by provoking the symptoms of nTOS. In support of feasibility for clinical translation, each of the nTOS patients in our cohort was able to complete this task. Using an inertial measurement unit placed on the upper extremity, we defined 18 kinetic and kinematic digital biomarkers of upper extremity motor capacity. We investigated which of these biomarkers could be useful for the clinical assessment of nTOS patients by identifying which discriminated the affected extremity of nTOS patients from their unaffected extremity, and from the extremities of healthy volunteers. Digital biomarkers representative of slowness, weakness, and rigidity were statistically different between the affected and unaffected extremities of patients with nTOS (six digital biomarkers) and between nTOS-affected and control extremities (nine digital biomarkers). We reasoned that digital biomarkers that are highly discriminatory in these analyses would be well-positioned to quantify nTOS disability. With particularly large effect sizes, speed and power emerged as candidate digital biomarkers.

To explore whether digital biomarkers of upper extremity motor capacity could have a clinical advantage over patient-reported measures, we investigated their association with work disability. We found that digital biomarkers of speed, rigidity, and exhaustion statistically differentiated patients reporting work disability from those that did not, whereas none of the clinical variables, demographic characteristics, or PROMs were able to do so. We also explored the ability of digital biomarkers to predict response to PT, as such a test would be useful in clinical practice to triage patients to best therapy and to limit periods of unproductive therapy. In line with prior reports from several groups [10,11,12,13,14,15,16], PT was unsuccessful in only 50% of the patients in our sample. While response to PT was not statistically correlated with any clinical or demographic variable, digital biomarkers of exhaustion of the affected arm (decline in power and increase in rise time) were significantly different between responders and non-responders. These data indicate that the objective assessment of nTOS-specific extremity function could provide clinically meaningful assessments of nTOS disability.

To explore whether digital biomarkers of nTOS disability defined by a single inertial measurement unit could be useful in addressing the challenges of monitoring response to nTOS therapy, we investigated a subset of four nTOS patients who underwent first rib resection and scalenectomy. In a Press Test performed one month after surgery, each of 18 digital biomarkers improved when compared with their preoperative assessment and biomarkers of speed, power, and exhaustion improved at higher rates than PROMs of DASH and VAS. These data demonstrate that digital biomarkers of upper extremity motor capacity could be useful for the objective assessment of nTOS therapies and may be more sensitive to change than patient-reported outcomes.

In addition to its clinical potential, sensor-based assessment of extremity function in nTOS provides unique insight into this condition that cannot be obtained from patient reports. For example, our data showed that the functional impairment of unilateral nTOS extended to the contralateral extremity. Nine digital biomarkers of slowness, weakness, and rigidity were statistically worse in the contralateral extremity of nTOS patients compared with the extremities of healthy individuals. Interestingly, these same digital biomarkers improved in the contralateral extremity following surgery and suggest that effective therapy for nTOS could also improve the functional performance of the “unaffected” side.

The strengths of this study include its prospective design and the development of novel sensor-based biomarkers of nTOS disability. Limitations include the exploratory nature of the clinical application of these biomarkers, such as prediction of response to PT and of monitoring of response to surgery, both of which will require validation in future studies. Additionally, compared with control patients, the nTOS group was enriched for females and for individuals with higher BMI; however, this was at least somewhat addressed by a sensitivity analysis that did not detect statistical differences in any of the 18 digital biomarkers between male and female control patients, and through statistical analyses that controlled for sex and BMI.

## 5. Conclusions

Digital biomarkers of disability, measurable using a single wearable sensor, are a promising, innovative platform for objectively assessing disability in nTOS. Our data support future investigation of candidate digital biomarkers including speed, power, and exhaustion to address major gaps in the field of nTOS, including predicting and monitoring responses to therapy. Such individualized markers of motor capacity could personalize the treatment of patients with nTOS by setting functional expectations and designing management strategies tailored to the recreational and work activity requirements of an individual. Perhaps most importantly, digital biomarkers of nTOS severity can provide the much needed framework of outcome metrics for clinical trials needed to advance the field.

## 6. Patents

Some of the results and methodologies described in this study were protected by a patent pending by the Baylor College of Medicine.

## Figures and Tables

**Table 1:** Patient Cohort.

	nTOS (n = 16)	Control (n = 13)	*p*-Value
Demographics			
Age, years	36.3 ± 12.9	30.2 ± 9.1	0.163
Female, n (%)	16 (100%)	5 (38%)	**<0.001**
Height, cm	162.8 ± 5.6	164.2 ± 10.1	0.717
Weight, kg	76.8 ± 14.8	64.5 ± 7.8	**0.030**
Body mass index, kg/m^2^	29.1 ± 4.5	24.5 ± 4.2	**0.016**
Right hand dominance, n (%)	13 (81%)	12 (92%)	0.606
Ethnicity, n (%) ** 0.008 **			
Caucasian	10 (63%)	5 (38%)	
African American	0	0	
Hispanic	1 (6%)	0	
Asian	1 (6%)	8 (62%)	
Declined reporting	4 (25%)	0	
Clinical characteristics			
nTOS in dominant arm, n (%)	10 (63%)	na	na
Duration of nTOS diagnosis, years	3.6 ± 4.9	na	na
Pain characteristics			
DASH	54.5 ± 25.7	1.80 ± 4.8	**<0.001**
VAS	5.4 ± 2.9	0 ± 0	**<0.001**
Headache, n (%)	7 (43%)	0 (0%)	**0.006**
Non-narcotic pain medication, n (%)	12 (75%)	0 (0%)	**<0.001**
Narcotics, n (%)	4 (25%)	na	na
Depression, CES-D	18.7 ± 12.4	na	na
Depressed, n (%)	7 (44%)	na	na
Limitation in ADL			
Barthel ADL Index	77.2 ± 34	100 ± 0	**<0.001**
Moderate/total dependence, n (%)	6 (38%)	0 (0%)	**0.017**
Moderate/severe ADL limitation, n (%)	12 (75%)	0 (0%)	**<0.001**
Work disability, n (%)	4 (25%)	0 (0%)	0.152

Mean ± standard deviation is shown unless otherwise noted. na: not applicable; VAS: visual analog scale (0–10); ADL: activities of daily living; CES-D: Center for Epidemiologic Studies Depression.

**Table 2:** Sensor-derived digital biomarkers of upper extremity motor capacity.

CATEGORY	DIGITAL BIOMARKER	DEFINITION
**Slowness**	Speed	Elbow angular velocity range
	Rise time	Duration of abduction acceleration
	Fall time	Duration of adduction acceleration
	Abduction time	Duration for arm rising from Position 1 to Position 2 (Figure 2)
	Adduction time	Duration for arm returning from Position 2 to Position 1 (Figure 2)
	Abduction+adduction time	Total duration for a cycle of abduction and adduction
	No. of cycles	Number of abduction+adduction repetitions per 20 s
**Weakness**	Power	Product of angular acceleration range and angular velocity range
**Rigidity**	Range of motion	Range of abduction/adduction rotation
**Exhaustion**	Decline in speed	Difference between the first and last 10 s of angular velocity
	Decline in power	Difference between the first and last 10 s of power
	Increase in abduction/adduction time	Difference between the first and last 10 s of abduction/adduction time
	Increase in rise time	Difference between the first and last 10 s of rise time duration
**Unsteadiness**	Speed variability	Coefficient of variation (CV) of speed
	Rise time variability	CV of rise time
	Abduction+adduction variability	CV of abduction+adduction time
	Power variability	CV of power
	Rigidity variability	CV of rigidity

**Table 3:** **Digital biomarkers of nTOS severity.** Affected and unaffected extremities of nTOS patients were compared. Shown are the variables that achieved statistical significance. Values for the dominant extremity of the control group are included as reference healthy benchmarks.

	Affected	Unaffected	p-Value *	Effect Size **	Control Group ^†^
**Slowness**					
Speed (deg/s)	218.4 ± 91	322.4 ± 107.1	<0.001	1.05	898.3 ± 240.8
Abduction time (msec)	621.1 ± 461	398 ± 97.7	0.023	0.69	213.9 ± 46.3
Abd/add time (msec)	1186.5 ± 813.6	798.3 ± 300	0.039	0.63	350.0 ± 99.4
# cycles (n)	9.8 ± 5.0	12.6 ± 3.7	<0.001	0.64	30.4 ± 9.8
**Weakness**					
Power (deg2/s3)	9358 ± 9361	20,519 ± 19,973	0.012	0.72	130,450 ± 86,355
**Rigidity**					
RoM (deg)	60.4 ± 17.9	72.1 ± 16.4	0.022	0.68	88.8 ± 27.3

* Cohen d effect size; ** Results adjusted for age and BMI; ^†^ Values for dominant extremity; RoM: Range of motion; Abd: Abduction; Add: Adduction; # cycles: number abduction/adduction cycles during 20 s; msec: milliseconds; s: second; deg: degree.

## Data Availability

Not applicable.
